# Flunarizinium hydrogen maleate

**DOI:** 10.1107/S1600536813020291

**Published:** 2013-07-27

**Authors:** Channappa N. Kavitha, Jerry P. Jasinski, Somer M. Matar, H. S. Yathirajan, A. R. Ramesha

**Affiliations:** a Department of Studies in Chemistry, University of Mysore, Manasagangotri, Mysore 570 006, India; b Department of Chemistry, Keene State College, 229 Main Street, Keene, NH 03435-2001, USA; c R. L. Fine Chem., Bangalore 560 064, India

## Abstract

In the cation of the title salt {systematic name: 4-[bis­(4-fluoro­phen­yl)meth­yl]-1-[(2*E*)-3-phenyl­prop-2-en-1-yl]piperazin-1-ium hydrogen maleate}, C_26_H_27_F_2_N_2_^+^·C_4_H_3_O_4_^−^, the protonated piperazine ring is in a chair conformation. The dihedral angle between the 4-fluoro­phenyl rings is 68.2 (2)°. An intra­molecular O—H⋯O hydrogen bond occurs in the anion. In the crystal, N—H⋯O, C—H⋯O and C—H⋯F inter­actions are observed, which link the ions into [001] chains.

## Related literature

For backgorund to flunarizine, see: Amery (1983 ▶); Holmes *et al.* (1984 ▶). For related structures, see: Jasinski, Butcher *et al.* (2010 ▶); Jasinski, Pek *et al.* (2010 ▶); Kavitha *et al.* (2013 ▶). For standard bond lengths, see: Allen *et al.* (1987 ▶).
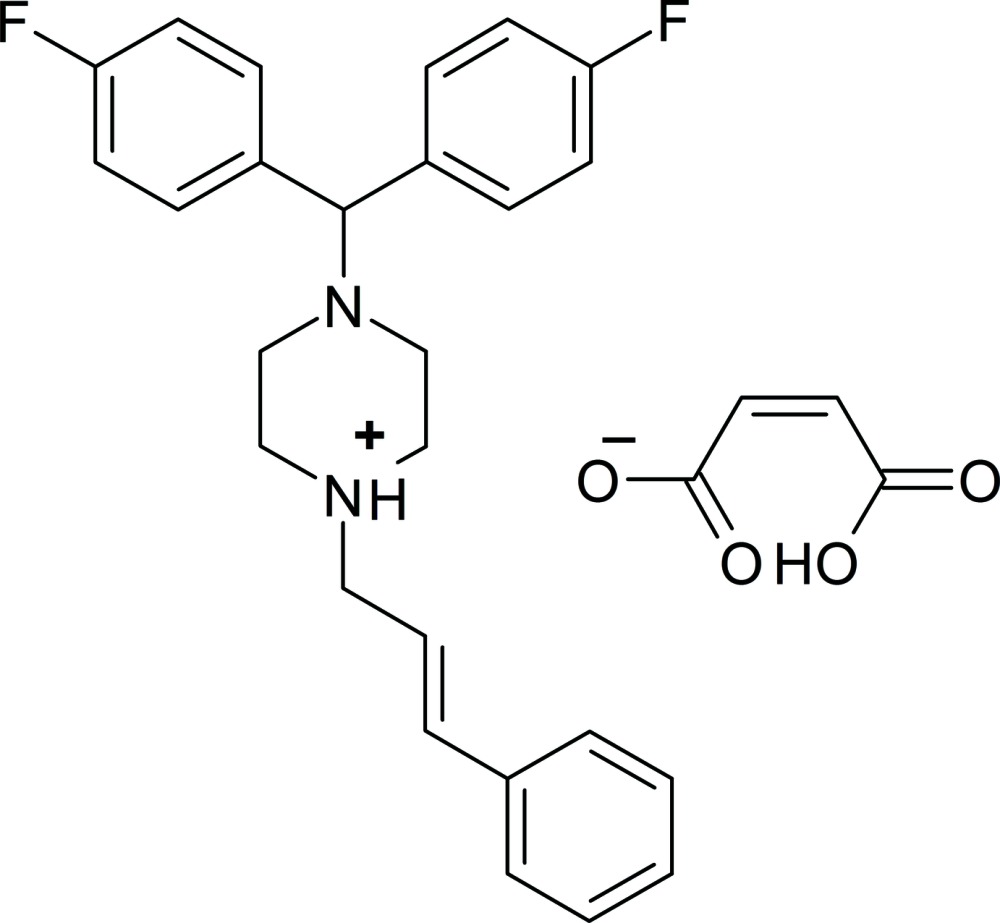


## Experimental

### 

#### Crystal data

C_26_H_27_F_2_N_2_^+^·C_4_H_3_O_4_^−^
*M* *_r_* = 520.56
Monoclinic, 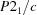

*a* = 22.1215 (5) Å
*b* = 10.8620 (2) Å
*c* = 11.3215 (2) Å
β = 98.879 (2)°
*V* = 2687.77 (9) Å^3^
*Z* = 4
Cu *K*α radiation
μ = 0.79 mm^−1^
*T* = 173 K
0.42 × 0.38 × 0.26 mm

#### Data collection

Agilent Xcalibur (Eos, Gemini) diffractometer
Absorption correction: multi-scan (*CrysAlis PRO* and *CrysAlis RED*; Agilent, 2012 ▶) *T*_min_ = 0.871, *T*_max_ = 1.000
17207 measured reflections
5260 independent reflections
4484 reflections with *I* > 2σ(*I*)
*R*_int_ = 0.040

#### Refinement

*R*[*F* ^2^ > 2σ(*F* ^2^)] = 0.048
*wR*(*F* ^2^) = 0.136
*S* = 1.03
5260 reflections
344 parameters
H-atom parameters constrained
Δρ_max_ = 0.52 e Å^−3^
Δρ_min_ = −0.22 e Å^−3^

### 

Data collection: *CrysAlis PRO* (Agilent, 2012 ▶); cell refinement: *CrysAlis PRO*; data reduction: *CrysAlis RED* (Agilent, 2012 ▶); program(s) used to solve structure: *SUPERFLIP* (Palatinus & Chapuis, 2007 ▶); program(s) used to refine structure: *SHELXL2012* (Sheldrick, 2008 ▶); molecular graphics: *OLEX2* (Dolomanov *et al.*, 2009 ▶); software used to prepare material for publication: *OLEX2*.

## Supplementary Material

Crystal structure: contains datablock(s) I. DOI: 10.1107/S1600536813020291/hb7110sup1.cif

Structure factors: contains datablock(s) I. DOI: 10.1107/S1600536813020291/hb7110Isup2.hkl

Supplementary material file. DOI: 10.1107/S1600536813020291/hb7110Isup3.cml

Additional supplementary materials:  crystallographic information; 3D view; checkCIF report

## Figures and Tables

**Table 1:** Hydrogen-bond geometry (Å, °)

*D*—H⋯*A*	*D*—H	H⋯*A*	*D*⋯*A*	*D*—H⋯*A*
O1*S*—H1*S*⋯O4*S*	0.82	1.63	2.451 (2)	177
N1—H1⋯O3*S*	0.91	1.83	2.7190 (18)	165
C1—H1*B*⋯O2*S* ^i^	0.97	2.51	3.354 (2)	146
C26—H26⋯O3*S* ^ii^	0.93	2.53	3.278 (2)	138
C2*S*—H2*S*⋯O4*S* ^iii^	0.93	2.46	3.386 (2)	171
C23—H23⋯F1^iv^	0.93	2.53	3.342 (2)	145

Symmetry codes: (i) 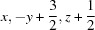; (ii) 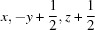; (iii) 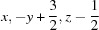; (iv) 
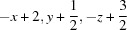
.

**Fig. 1.**
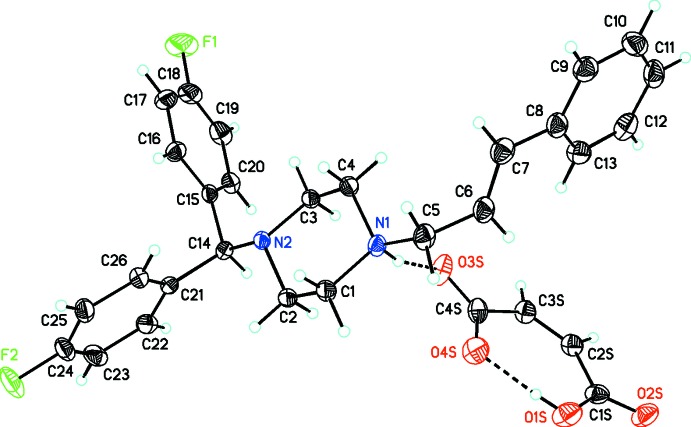
View of the asymmetric unit of (I) showing 30% probability displacement ellipsoids. Dashed lines indicate N1—H1···O3S intermolecular and O1S—H1S···O4S intramolecular hydrogen bond interactions.

**Fig. 2.**
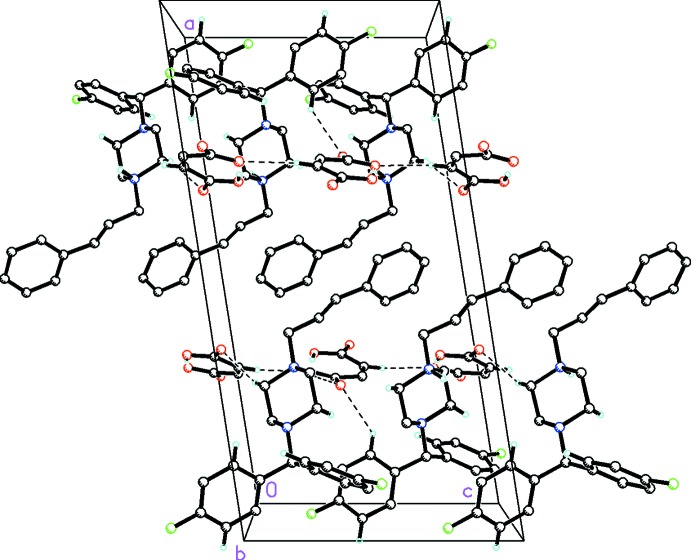
Molecular packing for (I) viewed along the b axis. Dashed lines indicate inter and intra molecular hydrogen bonds and weak C—H···O interactions linking the ions into [100] chains.

## supplementary crystallographic information

### Comment

Flunarizine (chemically, 1-[bis(4-fluorophenyl)methyl]-4-[(2E)-3-phenyl prop-2-en-1-yl]piperazine), a piperazine derivative is a non-selective calcium antagonist (Amery, 1983). A review of its pharmacodynamic and pharmacokinetic properties and therapeutic use is published (Holmes *et al.*, 1984).

In addition to the structures of trimipraminium maleate (Jasinski, Butcher *et al.*, 2010) and 4-(4-chlorophenyl)-4-hydroxypiperidinium maleate maleic acid solvate (Jasinski, Pek *et al.*, 2010), we have recently reported the crystal structure of 4-[bis(4-fluorophenyl) methyl]-1-[(2E)-3-phenylprop-2-en-1-yl]piperazin-1-ium 3-carboxy propanoate (Kavitha *et al.*, 2013). As part of our ongoing studies of molecular salts of bioactvive molecules, the paper reports the crystal and molecular structure of the title salt, (I).

The title compound, [systematic name: 1-[bis(4-fluorophenyl)methyl]-4- [(2E)-3-phenylprop-2-en-1-yl]piperazinium maleate], a maleate salt of Flunarizine crystallizes with one independent cation-anion pair in the asymmetric unit (Fig. 1). In the cation, the protonated piperazine ring is in a chair conformation (puckering parameters Q, θ, and φ = 0.5997 (16)Å, 179.21 (15)° and 65 (10)°, respectively). The dihedral angle between the mean planes of the 4-fluorophenyl rings is 68.2 (2)°. The extended phenyl ring is twisted by 15.8 (9)° and 59.8 (5)°, respectively, from these two rings. Bond lengths are in normal ranges (Allen *et al.*, 1987). Strong intramolecular O—H···O and intermolecular N—H···O hydrogen bonds and weak N—H···O, C—H···O, C—H···F intermolecular interactions (Table 1) are observed which link the ions into chains along [001] (Fig. 2).

### Experimental

Flunarizine (4.05 g, 0.01 mol) and maleic acid (1.16 g, 0.01 mol) were dissolved in hot N,N-dimethylformamide solution and stirred over a heating magnetic stirrer for 10 minutes. The resulting solution was allowed to cool slowly at room temperature. Colourless irregular crystals of the title compound (m. p.: 428– 433 K) appeared after a few days.

### Refinement

H1 and H1S were located by a difference map and refined isotropically. All of the remaining H atoms were placed in their calculated positions and then refined using the riding model with Atom—H lengths of 0.93Å, 0.98Å (CH) or 0.97Å (CH_2_). Isotropic displacement parameters for these atoms were set to 1.2 (CH, CH_2_) or 1.5 (OH) times *U*_eq_ of the parent atom.

### Crystal data

**Table:**

C_26_H_27_F_2_N_2_^+^·C_4_H_3_O_4_^−^	*F*(000) = 1096
*M**_r_* = 520.56	*D*_x_ = 1.286 Mg m^−^^3^
Monoclinic, *P*2_1_/*c*	Cu *K*α radiation, λ = 1.5418 Å
*a* = 22.1215 (5) Å	Cell parameters from 6479 reflections
*b* = 10.8620 (2) Å	θ = 4.0–72.3°
*c* = 11.3215 (2) Å	µ = 0.79 mm^−^^1^
β = 98.879 (2)°	*T* = 173 K
*V* = 2687.77 (9) Å^3^	Irregular, colourless
*Z* = 4	0.42 × 0.38 × 0.26 mm

### Data collection

**Table:**

Agilent Xcalibur (Eos, Gemini) diffractometer	5260 independent reflections
Radiation source: Enhance (Cu) X-ray Source	4484 reflections with *I* > 2σ(*I*)
Graphite monochromator	*R*_int_ = 0.040
Detector resolution: 16.0416 pixels mm^-1^	θ_max_ = 72.5°, θ_min_ = 4.1°
ω scans	*h* = −24→27
Absorption correction: multi-scan (*CrysAlis PRO* and *CrysAlis RED*; Agilent, 2012)	*k* = −12→13
*T*_min_ = 0.871, *T*_max_ = 1.000	*l* = −13→8
17207 measured reflections	

### Refinement

**Table:**

Refinement on *F*^2^	Hydrogen site location: inferred from neighbouring sites
Least-squares matrix: full	H-atom parameters constrained
*R*[*F*^2^ > 2σ(*F*^2^)] = 0.048	*w* = 1/[σ^2^(*F*_o_^2^) + (0.0702*P*)^2^ + 0.7817*P*] where *P* = (*F*_o_^2^ + 2*F*_c_^2^)/3
*wR*(*F*^2^) = 0.136	(Δ/σ)_max_ = 0.001
*S* = 1.03	Δρ_max_ = 0.52 e Å^−^^3^
5260 reflections	Δρ_min_ = −0.22 e Å^−^^3^
344 parameters	Extinction correction: *SHELXL2012* (Sheldrick, 2008), Fc^*^=kFc[1+0.001xFc^2^λ^3^/sin(2θ)]^-1/4^
0 restraints	Extinction coefficient: 0.0021 (2)
Primary atom site location: structure-invariant direct methods	

### Special details

**Table:**

Geometry. All esds (except the esd in the dihedral angle between two l.s. planes) are estimated using the full covariance matrix. The cell esds are taken into account individually in the estimation of esds in distances, angles and torsion angles; correlations between esds in cell parameters are only used when they are defined by crystal symmetry. An approximate (isotropic) treatment of cell esds is used for estimating esds involving l.s. planes.

### Fractional atomic coordinates and isotropic or equivalent isotropic displacement parameters (Å^2^)

**Table:**

	*x*	*y*	*z*	*U*_iso_*/*U*_eq_	
F1	0.87928 (7)	−0.24891 (14)	0.47740 (13)	0.0848 (5)	
F2	0.98166 (6)	0.06251 (14)	1.26090 (10)	0.0727 (4)	
N1	0.68788 (6)	0.32014 (12)	0.73573 (11)	0.0319 (3)	
H1	0.7039	0.3791	0.6926	0.038*	
N2	0.79790 (5)	0.17520 (11)	0.79037 (10)	0.0280 (3)	
C1	0.72463 (8)	0.31929 (15)	0.85715 (13)	0.0363 (4)	
H1A	0.7093	0.2563	0.9055	0.044*	
H1B	0.7211	0.3983	0.8954	0.044*	
C2	0.79108 (7)	0.29391 (14)	0.84796 (13)	0.0332 (3)	
H2A	0.8066	0.3588	0.8021	0.040*	
H2B	0.8150	0.2938	0.9274	0.040*	
C3	0.76221 (7)	0.17749 (14)	0.67002 (12)	0.0306 (3)	
H3A	0.7669	0.0997	0.6303	0.037*	
H3B	0.7773	0.2425	0.6236	0.037*	
C4	0.69533 (7)	0.19923 (14)	0.67663 (13)	0.0330 (3)	
H4A	0.6720	0.1992	0.5967	0.040*	
H4B	0.6799	0.1336	0.7218	0.040*	
C5	0.62138 (8)	0.35276 (17)	0.73677 (15)	0.0411 (4)	
H5A	0.5996	0.2815	0.7601	0.049*	
H5B	0.6185	0.4179	0.7942	0.049*	
C6	0.59335 (8)	0.39422 (18)	0.61479 (16)	0.0445 (4)	
H6	0.6034	0.4726	0.5911	0.053*	
C7	0.55601 (8)	0.32949 (17)	0.53891 (18)	0.0470 (4)	
H7	0.5435	0.2535	0.5645	0.056*	
C8	0.53185 (8)	0.36771 (17)	0.41482 (16)	0.0433 (4)	
C9	0.47868 (9)	0.31385 (18)	0.35792 (19)	0.0496 (4)	
H9	0.4588	0.2557	0.3984	0.059*	
C10	0.45461 (9)	0.3450 (2)	0.2417 (2)	0.0579 (5)	
H10	0.4186	0.3083	0.2051	0.069*	
C11	0.48338 (10)	0.4293 (2)	0.18018 (18)	0.0564 (5)	
H11	0.4669	0.4505	0.1023	0.068*	
C12	0.53710 (10)	0.48277 (19)	0.23452 (19)	0.0565 (5)	
H12	0.5573	0.5392	0.1927	0.068*	
C13	0.56108 (9)	0.45259 (18)	0.35134 (18)	0.0501 (4)	
H13	0.5971	0.4896	0.3876	0.060*	
C14	0.86369 (7)	0.14809 (13)	0.79043 (13)	0.0287 (3)	
H14	0.8825	0.2201	0.7587	0.034*	
C15	0.87072 (6)	0.03898 (14)	0.70972 (13)	0.0292 (3)	
C16	0.84983 (8)	−0.07710 (15)	0.73468 (15)	0.0378 (4)	
H16	0.8335	−0.0896	0.8046	0.045*	
C17	0.85290 (9)	−0.17466 (17)	0.65714 (18)	0.0486 (4)	
H17	0.8386	−0.2523	0.6738	0.058*	
C18	0.87752 (9)	−0.15360 (19)	0.55528 (18)	0.0528 (5)	
C19	0.90001 (9)	−0.0420 (2)	0.52802 (16)	0.0502 (5)	
H19	0.9173	−0.0313	0.4589	0.060*	
C20	0.89645 (7)	0.05539 (16)	0.60644 (14)	0.0377 (4)	
H20	0.9115	0.1323	0.5895	0.045*	
C21	0.89532 (7)	0.12623 (13)	0.91779 (13)	0.0298 (3)	
C22	0.95568 (7)	0.16234 (15)	0.95284 (15)	0.0384 (4)	
H22	0.9765	0.2017	0.8983	0.046*	
C23	0.98534 (8)	0.14033 (19)	1.06856 (17)	0.0490 (5)	
H23	1.0258	0.1644	1.0919	0.059*	
C24	0.95369 (9)	0.08262 (18)	1.14703 (15)	0.0468 (4)	
C25	0.89418 (8)	0.04536 (16)	1.11636 (15)	0.0419 (4)	
H25	0.8738	0.0058	1.1716	0.050*	
C26	0.86487 (7)	0.06801 (15)	1.00075 (13)	0.0345 (3)	
H26	0.8244	0.0439	0.9786	0.041*	
O1S	0.67312 (8)	0.83923 (13)	0.66302 (12)	0.0604 (4)	
H1S	0.6848	0.7679	0.6735	0.091*	
O2S	0.65899 (8)	0.97228 (12)	0.51543 (12)	0.0589 (4)	
O3S	0.72834 (6)	0.47153 (13)	0.57335 (13)	0.0561 (4)	
O4S	0.70541 (8)	0.62367 (13)	0.68757 (11)	0.0600 (4)	
C1S	0.67577 (8)	0.87144 (16)	0.55309 (15)	0.0411 (4)	
C2S	0.70000 (8)	0.78300 (16)	0.47107 (14)	0.0400 (4)	
H2S	0.7043	0.8161	0.3971	0.048*	
C3S	0.71654 (8)	0.66586 (16)	0.48435 (15)	0.0406 (4)	
H3S	0.7298	0.6303	0.4182	0.049*	
C4S	0.71709 (8)	0.58175 (17)	0.58972 (16)	0.0410 (4)	

### Atomic displacement parameters (Å^2^)

**Table:**

	*U*^11^	*U*^22^	*U*^33^	*U*^12^	*U*^13^	*U*^23^
F1	0.0965 (11)	0.0745 (9)	0.0831 (10)	0.0137 (8)	0.0126 (8)	−0.0504 (8)
F2	0.0663 (8)	0.1044 (11)	0.0390 (6)	0.0294 (7)	−0.0186 (5)	−0.0032 (6)
N1	0.0355 (7)	0.0320 (7)	0.0284 (6)	0.0097 (5)	0.0059 (5)	0.0009 (5)
N2	0.0315 (6)	0.0274 (6)	0.0241 (6)	0.0060 (5)	0.0014 (5)	−0.0013 (5)
C1	0.0445 (9)	0.0383 (8)	0.0256 (7)	0.0111 (7)	0.0034 (6)	−0.0052 (6)
C2	0.0402 (8)	0.0310 (8)	0.0271 (7)	0.0067 (6)	0.0008 (6)	−0.0050 (6)
C3	0.0352 (8)	0.0316 (7)	0.0241 (7)	0.0072 (6)	0.0020 (6)	−0.0041 (5)
C4	0.0351 (8)	0.0330 (8)	0.0297 (8)	0.0076 (6)	0.0010 (6)	−0.0045 (6)
C5	0.0374 (9)	0.0461 (9)	0.0414 (9)	0.0136 (7)	0.0108 (7)	−0.0006 (7)
C6	0.0380 (9)	0.0456 (10)	0.0499 (10)	0.0139 (7)	0.0062 (8)	−0.0011 (8)
C7	0.0431 (10)	0.0419 (10)	0.0575 (11)	0.0018 (7)	0.0128 (8)	0.0039 (8)
C8	0.0402 (9)	0.0437 (9)	0.0462 (10)	0.0112 (7)	0.0071 (7)	−0.0011 (7)
C9	0.0452 (10)	0.0438 (10)	0.0606 (12)	0.0033 (8)	0.0110 (9)	−0.0039 (8)
C10	0.0440 (10)	0.0640 (13)	0.0622 (12)	0.0092 (9)	−0.0027 (9)	−0.0222 (10)
C11	0.0629 (13)	0.0612 (13)	0.0429 (10)	0.0279 (10)	0.0016 (9)	−0.0051 (9)
C12	0.0714 (14)	0.0420 (10)	0.0583 (12)	0.0101 (9)	0.0170 (10)	0.0076 (9)
C13	0.0463 (10)	0.0471 (10)	0.0557 (11)	−0.0010 (8)	0.0040 (8)	−0.0049 (8)
C14	0.0313 (7)	0.0256 (7)	0.0296 (7)	0.0016 (5)	0.0058 (6)	0.0018 (5)
C15	0.0284 (7)	0.0304 (7)	0.0276 (7)	0.0068 (6)	0.0003 (5)	0.0003 (6)
C16	0.0425 (9)	0.0316 (8)	0.0394 (8)	0.0040 (7)	0.0070 (7)	−0.0025 (6)
C17	0.0504 (10)	0.0337 (9)	0.0591 (11)	0.0052 (7)	0.0007 (9)	−0.0101 (8)
C18	0.0516 (11)	0.0530 (11)	0.0509 (11)	0.0156 (9)	−0.0015 (8)	−0.0254 (9)
C19	0.0497 (10)	0.0682 (13)	0.0337 (9)	0.0147 (9)	0.0092 (7)	−0.0096 (8)
C20	0.0370 (8)	0.0439 (9)	0.0324 (8)	0.0065 (7)	0.0054 (6)	0.0021 (7)
C21	0.0318 (7)	0.0256 (7)	0.0311 (7)	0.0045 (6)	0.0018 (6)	−0.0035 (6)
C22	0.0319 (8)	0.0391 (9)	0.0434 (9)	0.0023 (6)	0.0040 (7)	−0.0071 (7)
C23	0.0321 (8)	0.0586 (11)	0.0520 (10)	0.0084 (8)	−0.0072 (8)	−0.0149 (9)
C24	0.0474 (10)	0.0564 (11)	0.0319 (8)	0.0223 (8)	−0.0090 (7)	−0.0075 (7)
C25	0.0505 (10)	0.0432 (9)	0.0310 (8)	0.0126 (7)	0.0034 (7)	0.0023 (7)
C26	0.0358 (8)	0.0350 (8)	0.0313 (8)	0.0020 (6)	0.0003 (6)	0.0007 (6)
O1S	0.0998 (12)	0.0472 (8)	0.0383 (7)	−0.0015 (7)	0.0241 (7)	−0.0015 (6)
O2S	0.0891 (11)	0.0335 (7)	0.0551 (8)	−0.0014 (7)	0.0143 (7)	0.0013 (6)
O3S	0.0599 (8)	0.0490 (8)	0.0651 (9)	0.0202 (6)	0.0275 (7)	0.0206 (6)
O4S	0.0962 (12)	0.0536 (8)	0.0317 (6)	0.0022 (8)	0.0142 (7)	0.0111 (6)
C1S	0.0502 (10)	0.0368 (9)	0.0362 (8)	−0.0123 (7)	0.0061 (7)	−0.0007 (7)
C2S	0.0497 (10)	0.0432 (9)	0.0278 (8)	−0.0045 (7)	0.0084 (7)	0.0065 (7)
C3S	0.0455 (9)	0.0461 (10)	0.0323 (8)	0.0034 (7)	0.0122 (7)	0.0056 (7)
C4S	0.0361 (8)	0.0469 (10)	0.0411 (9)	0.0041 (7)	0.0092 (7)	0.0126 (7)

### Geometric parameters (Å, º)

**Table:**

F1—C18	1.364 (2)	C12—C13	1.386 (3)
F2—C24	1.3589 (19)	C13—H13	0.9300
N1—H1	0.9101	C14—H14	0.9800
N1—C1	1.4851 (19)	C14—C15	1.519 (2)
N1—C4	1.4945 (18)	C14—C21	1.521 (2)
N1—C5	1.5150 (19)	C15—C16	1.387 (2)
N2—C2	1.4632 (18)	C15—C20	1.389 (2)
N2—C3	1.4657 (17)	C16—H16	0.9300
N2—C14	1.4848 (18)	C16—C17	1.384 (2)
C1—H1A	0.9700	C17—H17	0.9300
C1—H1B	0.9700	C17—C18	1.368 (3)
C1—C2	1.515 (2)	C18—C19	1.364 (3)
C2—H2A	0.9700	C19—H19	0.9300
C2—H2B	0.9700	C19—C20	1.391 (2)
C3—H3A	0.9700	C20—H20	0.9300
C3—H3B	0.9700	C21—C22	1.390 (2)
C3—C4	1.512 (2)	C21—C26	1.390 (2)
C4—H4A	0.9700	C22—H22	0.9300
C4—H4B	0.9700	C22—C23	1.392 (2)
C5—H5A	0.9700	C23—H23	0.9300
C5—H5B	0.9700	C23—C24	1.365 (3)
C5—C6	1.493 (2)	C24—C25	1.370 (3)
C6—H6	0.9300	C25—H25	0.9300
C6—C7	1.302 (3)	C25—C26	1.390 (2)
C7—H7	0.9300	C26—H26	0.9300
C7—C8	1.483 (3)	O1S—H1S	0.8199
C8—C9	1.381 (3)	O1S—C1S	1.303 (2)
C8—C13	1.389 (3)	O2S—C1S	1.213 (2)
C9—H9	0.9300	O3S—C4S	1.243 (2)
C9—C10	1.383 (3)	O4S—C4S	1.261 (2)
C10—H10	0.9300	C1S—C2S	1.492 (2)
C10—C11	1.366 (3)	C2S—H2S	0.9300
C11—H11	0.9300	C2S—C3S	1.326 (2)
C11—C12	1.379 (3)	C3S—H3S	0.9300
C12—H12	0.9300	C3S—C4S	1.501 (2)
			
C1—N1—H1	107.4	C13—C12—H12	119.9
C1—N1—C4	109.06 (11)	C8—C13—H13	119.6
C1—N1—C5	112.81 (12)	C12—C13—C8	120.71 (19)
C4—N1—H1	107.4	C12—C13—H13	119.6
C4—N1—C5	112.53 (13)	N2—C14—H14	108.3
C5—N1—H1	107.4	N2—C14—C15	110.23 (12)
C2—N2—C3	108.75 (11)	N2—C14—C21	109.81 (11)
C2—N2—C14	110.06 (12)	C15—C14—H14	108.3
C3—N2—C14	113.02 (11)	C15—C14—C21	111.94 (11)
N1—C1—H1A	109.7	C21—C14—H14	108.3
N1—C1—H1B	109.7	C16—C15—C14	121.22 (13)
N1—C1—C2	109.63 (12)	C16—C15—C20	118.75 (14)
H1A—C1—H1B	108.2	C20—C15—C14	119.98 (14)
C2—C1—H1A	109.7	C15—C16—H16	119.5
C2—C1—H1B	109.7	C17—C16—C15	121.07 (16)
N2—C2—C1	111.03 (13)	C17—C16—H16	119.5
N2—C2—H2A	109.4	C16—C17—H17	120.9
N2—C2—H2B	109.4	C18—C17—C16	118.12 (18)
C1—C2—H2A	109.4	C18—C17—H17	120.9
C1—C2—H2B	109.4	F1—C18—C17	118.2 (2)
H2A—C2—H2B	108.0	C19—C18—F1	118.71 (19)
N2—C3—H3A	109.6	C19—C18—C17	123.09 (16)
N2—C3—H3B	109.6	C18—C19—H19	120.9
N2—C3—C4	110.34 (11)	C18—C19—C20	118.22 (17)
H3A—C3—H3B	108.1	C20—C19—H19	120.9
C4—C3—H3A	109.6	C15—C20—C19	120.71 (17)
C4—C3—H3B	109.6	C15—C20—H20	119.6
N1—C4—C3	109.62 (12)	C19—C20—H20	119.6
N1—C4—H4A	109.7	C22—C21—C14	120.52 (14)
N1—C4—H4B	109.7	C26—C21—C14	120.78 (13)
C3—C4—H4A	109.7	C26—C21—C22	118.69 (14)
C3—C4—H4B	109.7	C21—C22—H22	119.6
H4A—C4—H4B	108.2	C21—C22—C23	120.84 (16)
N1—C5—H5A	109.8	C23—C22—H22	119.6
N1—C5—H5B	109.8	C22—C23—H23	120.8
H5A—C5—H5B	108.3	C24—C23—C22	118.47 (16)
C6—C5—N1	109.17 (13)	C24—C23—H23	120.8
C6—C5—H5A	109.8	F2—C24—C23	119.13 (17)
C6—C5—H5B	109.8	F2—C24—C25	118.18 (18)
C5—C6—H6	117.4	C23—C24—C25	122.68 (16)
C7—C6—C5	125.30 (19)	C24—C25—H25	120.8
C7—C6—H6	117.4	C24—C25—C26	118.47 (17)
C6—C7—H7	117.5	C26—C25—H25	120.8
C6—C7—C8	124.96 (18)	C21—C26—C25	120.85 (15)
C8—C7—H7	117.5	C21—C26—H26	119.6
C9—C8—C7	118.67 (18)	C25—C26—H26	119.6
C9—C8—C13	118.10 (18)	C1S—O1S—H1S	109.4
C13—C8—C7	123.21 (17)	O1S—C1S—C2S	119.68 (16)
C8—C9—H9	119.5	O2S—C1S—O1S	121.46 (17)
C8—C9—C10	121.01 (19)	O2S—C1S—C2S	118.86 (16)
C10—C9—H9	119.5	C1S—C2S—H2S	114.1
C9—C10—H10	119.7	C3S—C2S—C1S	131.80 (15)
C11—C10—C9	120.5 (2)	C3S—C2S—H2S	114.1
C11—C10—H10	119.7	C2S—C3S—H3S	115.0
C10—C11—H11	120.3	C2S—C3S—C4S	129.96 (16)
C10—C11—C12	119.48 (19)	C4S—C3S—H3S	115.0
C12—C11—H11	120.3	O3S—C4S—O4S	123.50 (16)
C11—C12—H12	119.9	O3S—C4S—C3S	116.43 (16)
C11—C12—C13	120.2 (2)	O4S—C4S—C3S	120.06 (16)
			
F1—C18—C19—C20	178.26 (17)	C13—C8—C9—C10	−1.1 (3)
F2—C24—C25—C26	178.65 (15)	C14—N2—C2—C1	−175.83 (12)
N1—C1—C2—N2	−59.35 (16)	C14—N2—C3—C4	177.21 (12)
N1—C5—C6—C7	104.8 (2)	C14—C15—C16—C17	175.99 (15)
N2—C3—C4—N1	60.33 (16)	C14—C15—C20—C19	−176.28 (15)
N2—C14—C15—C16	−63.63 (17)	C14—C21—C22—C23	−178.65 (14)
N2—C14—C15—C20	113.99 (15)	C14—C21—C26—C25	178.47 (14)
N2—C14—C21—C22	−145.85 (14)	C15—C14—C21—C22	91.37 (16)
N2—C14—C21—C26	35.24 (18)	C15—C14—C21—C26	−87.53 (17)
C1—N1—C4—C3	−58.52 (16)	C15—C16—C17—C18	0.4 (3)
C1—N1—C5—C6	160.42 (14)	C16—C15—C20—C19	1.4 (2)
C2—N2—C3—C4	−60.26 (16)	C16—C17—C18—F1	−178.50 (17)
C2—N2—C14—C15	−168.65 (11)	C16—C17—C18—C19	1.2 (3)
C2—N2—C14—C21	67.58 (14)	C17—C18—C19—C20	−1.4 (3)
C3—N2—C2—C1	59.86 (15)	C18—C19—C20—C15	0.1 (3)
C3—N2—C14—C15	−46.84 (15)	C20—C15—C16—C17	−1.7 (2)
C3—N2—C14—C21	−170.62 (12)	C21—C14—C15—C16	58.90 (18)
C4—N1—C1—C2	57.71 (16)	C21—C14—C15—C20	−123.48 (15)
C4—N1—C5—C6	−75.65 (17)	C21—C22—C23—C24	−0.1 (3)
C5—N1—C1—C2	−176.47 (13)	C22—C21—C26—C25	−0.5 (2)
C5—N1—C4—C3	175.51 (12)	C22—C23—C24—F2	−178.81 (15)
C5—C6—C7—C8	−175.39 (15)	C22—C23—C24—C25	0.2 (3)
C6—C7—C8—C9	−158.61 (19)	C23—C24—C25—C26	−0.4 (3)
C6—C7—C8—C13	23.4 (3)	C24—C25—C26—C21	0.5 (2)
C7—C8—C9—C10	−179.18 (17)	C26—C21—C22—C23	0.3 (2)
C7—C8—C13—C12	178.51 (17)	O1S—C1S—C2S—C3S	6.3 (3)
C8—C9—C10—C11	0.6 (3)	O2S—C1S—C2S—C3S	−173.7 (2)
C9—C8—C13—C12	0.5 (3)	C1S—C2S—C3S—C4S	−0.9 (3)
C9—C10—C11—C12	0.5 (3)	C2S—C3S—C4S—O3S	172.11 (19)
C10—C11—C12—C13	−1.1 (3)	C2S—C3S—C4S—O4S	−6.6 (3)
C11—C12—C13—C8	0.6 (3)		

### Hydrogen-bond geometry (Å, º)

**Table:**

*D*—H···*A*	*D*—H	H···*A*	*D*···*A*	*D*—H···*A*
O1*S*—H1*S*···O4*S*	0.82	1.63	2.451 (2)	177
N1—H1···O3*S*	0.91	1.83	2.7190 (18)	165
C1—H1*B*···O2*S*^i^	0.97	2.51	3.354 (2)	146
C26—H26···O3*S*^ii^	0.93	2.53	3.278 (2)	138
C2*S*—H2*S*···O4*S*^iii^	0.93	2.46	3.386 (2)	171
C23—H23···F1^iv^	0.93	2.53	3.342 (2)	145

Symmetry codes: (i) *x*, −*y*+3/2, *z*+1/2; (ii) *x*, −*y*+1/2, *z*+1/2; (iii) *x*, −*y*+3/2, *z*−1/2; (iv) −*x*+2, *y*+1/2, −*z*+3/2.
